# Factors influencing the acceptance of deceased donor kidney transplant offers in children—a vignette survey

**DOI:** 10.1093/ckj/sfag211

**Published:** 2026-06-22

**Authors:** Leonie Greipel, Xiaofei Liu, Evgenia Preka, Lena Brunkhorst, Burkhard Tönshoff, Dieter Haffner, Nele Kanzelmeyer

**Affiliations:** Department of Pediatric Kidney, Liver, Metabolic and Neurological Diseases, Hannover Medical School, Hannover, Germany; PRACTIS Clinician Scientist Program, Dean’s Office for Academic Career Development, Hannover Medical School, Hannover, Germany; Institute for Biostatistics, Hannover Medical School, Hannover, Germany; Université Paris Cité, INSERM U970 PARCC, Paris Institute for Transplantation and Organ Regeneration, Paris, France; Pediatric Nephrology, Necker Enfants Malades University Hospital, Université Paris Cité, Paris, France; Department of Pediatric Kidney, Liver, Metabolic and Neurological Diseases, Hannover Medical School, Hannover, Germany; Heidelberg University, Medical Faculty, Department of Pediatrics I, University Children’s Hospital Heidelberg, Heidelberg, Germany; Department of Pediatric Kidney, Liver, Metabolic and Neurological Diseases, Hannover Medical School, Hannover, Germany; Department of Pediatric Kidney, Liver, Metabolic and Neurological Diseases, Hannover Medical School, Hannover, Germany

**Keywords:** chronic renal failure, CKD, graft survival, kidney transplantation, pediatrics

## Abstract

**Background:**

Donor kidneys are allocated to potential pediatric kidney transplant recipients based on criteria such as human leukocyte antigen (HLA) immunology, but explicit guidance for accepting deceased-donor kidney offers is lacking.

**Methods:**

A vignette survey was designed with a matrix of 128 fictitious kidney offers consisting of seven dimensions with two levels each: mismatches on HLA A, B, C (≤3/≥4); mismatches on HLA DR, DQ (≤2/≥3); donor estimated glomerular filtration rate (eGFR) (≥50/≤49 ml/min/1.73 m^2^); donor age (≤1/2–50 years); recipient age (≤1/≥2 years); recipient status (preemptive/on dialysis); donor history of sepsis or bacterial infection (yes/no). Between December 2024 and February 2025, clinicians involved in pediatric kidney transplantation evaluated a randomly assigned set of 16 vignettes. Mixed-effects logistic regression assessed factors associated with acceptance.

**Results:**

Forty participants from eleven allocation organizations in Europe, Iran, and Canada, most commonly Eurotransplant (58%), including eight European countries, provided 533 evaluations (3–6 per vignette). In the multivariate analysis, four of the seven vignette dimensions decreased acceptance: ≥4 HLA A, B, C mismatches (*P* = .011), ≥3 HLA DR, DQ mismatches (*P* < .001), donor eGFR ≤49 ml/min/1.73 m^2^ (*P* < .001), or donor age ≤1 year (*P* = .003). Recipient age, dialysis status, and donor sepsis/bacterial infection were not significant. Allocation organization and respondent characteristics (gender and experience) did not materially influence decisions.

**Conclusions:**

This pilot study suggests that factors such as HLA mismatches, donor eGFR, and donor age, consistently guided decisions, independently of participant’s allocation organization. This underscores the potential value of developing evidence-informed donor-offer acceptance guidelines and investigating the impact of these factors on kidney transplantation outcome.

## Graphical Abstract

**Graphical Abstract:**
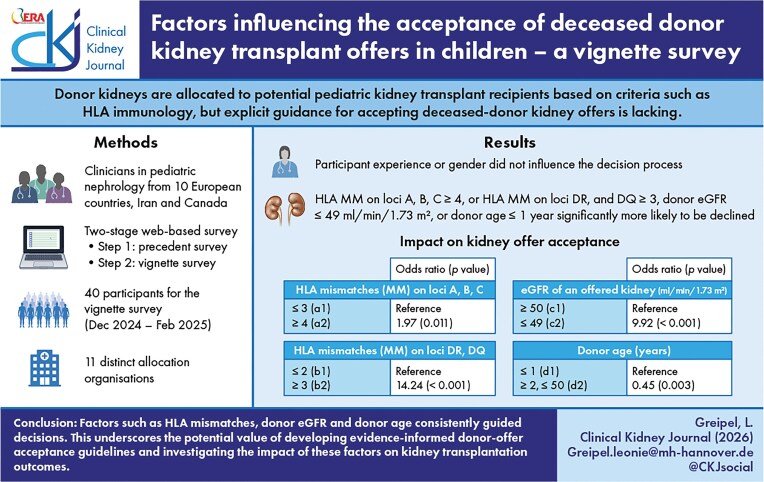

KEY LEARNING POINTS **What was known:**

- Deceased donor kidneys are allocated to potential pediatric kidney transplant recipients according to strict criteria defined by the kidney allocation system.
- If a deceased donor kidney is allocated to a specific pediatric transplant recipient, there are no universally accepted guidelines whether or not the donor organ should be accepted for this child by the local transplant center.
- The main aim of this study was to investigate the criteria that influence the decision-making process of accepting a kidney offer in children.

**This study adds:**

- In our factorial vignette survey among clinicians from 11 different allocation organizations, four of seven factors significantly impacted the kidney offer acceptance (higher HLA mismatches on HLA loci A, B, and C or DR and DQ, donor eGFR, and donor age).
- Three of seven dimensions, such as recipient age, recipient status (preemptive or on dialysis), and a donor with a history of sepsis or bacterial infection, had no significant influence on the acceptance of kidney offer.
- Allocation organization and respondent characteristics (gender and experience) did not materially influence decisions.

**Potential impact:**

- These results support the need for future investigations in this field and the development of evidence-based tools to aid in the decision-making process.
- Our findings suggest that there is potential for further research leading toward explicit donor acceptance guidelines in children.

## INTRODUCTION

Strict criteria for the allocation of organs from deceased donors determine the order in which potential recipients are offered a kidney transplant (KTx). The allocation criteria in the Eurotransplant Zone (Austria, Belgium, Croatia, Germany, Hungary, Luxembourg, the Netherlands, and Slovenia) include, e.g. human leukocyte antigen (HLA) immune status, blood type, and waiting time, with a pediatric bonus usually being granted [1, 2].

However, the acceptance of KTx offers varies widely among different nephrologists and centers [3, 4]. If a deceased donor kidney is allocated to a pediatric recipient, there are no universally accepted guidelines for acceptance by the local transplant center. Identifying the optimal offer is crucial, as graft longevity is limited and children often require multiple KTx given an estimated graft half-life of 12–15 years [5]. The outcome of KTx in children has improved over the last few decades [6]. A graft survival rate of 77.4% at 5 years and 66.5% at 10 years after KTx in children was reported in a European cohort between 1981 and 2016 [7]. In a large European cohort, between 2007 and 2015 the graft survival after KTx in patients younger than 20 years was 88% after 5 years [8]. Beyond short-term outcomes, choosing when to accept a specific offer can affect lifetime HLA sensitization, options for future grafts and cumulative exposure to dialysis [9].

To evaluate this complex decision-making process, we used a vignette-based survey design that allows simultaneous testing [10–12] of multiple, predefined attributes in standardized, realistic scenarios and has been applied to multifactorial clinical decisions, such as factors influencing KTx recommendations [13], and other clinical decision processes [14, 15].

This study aims to shed light on the complex decision-making process when accepting a KTx offer for pediatric patients in international transplant centers where these system-level rules still leave local teams to decide whether to accept a specific offer. To our knowledge we present the first vignette survey investigating factors contributing to the acceptance of a donor kidney offer in children.

## MATERIALS AND METHODS

### Study design

**Figure 1:**
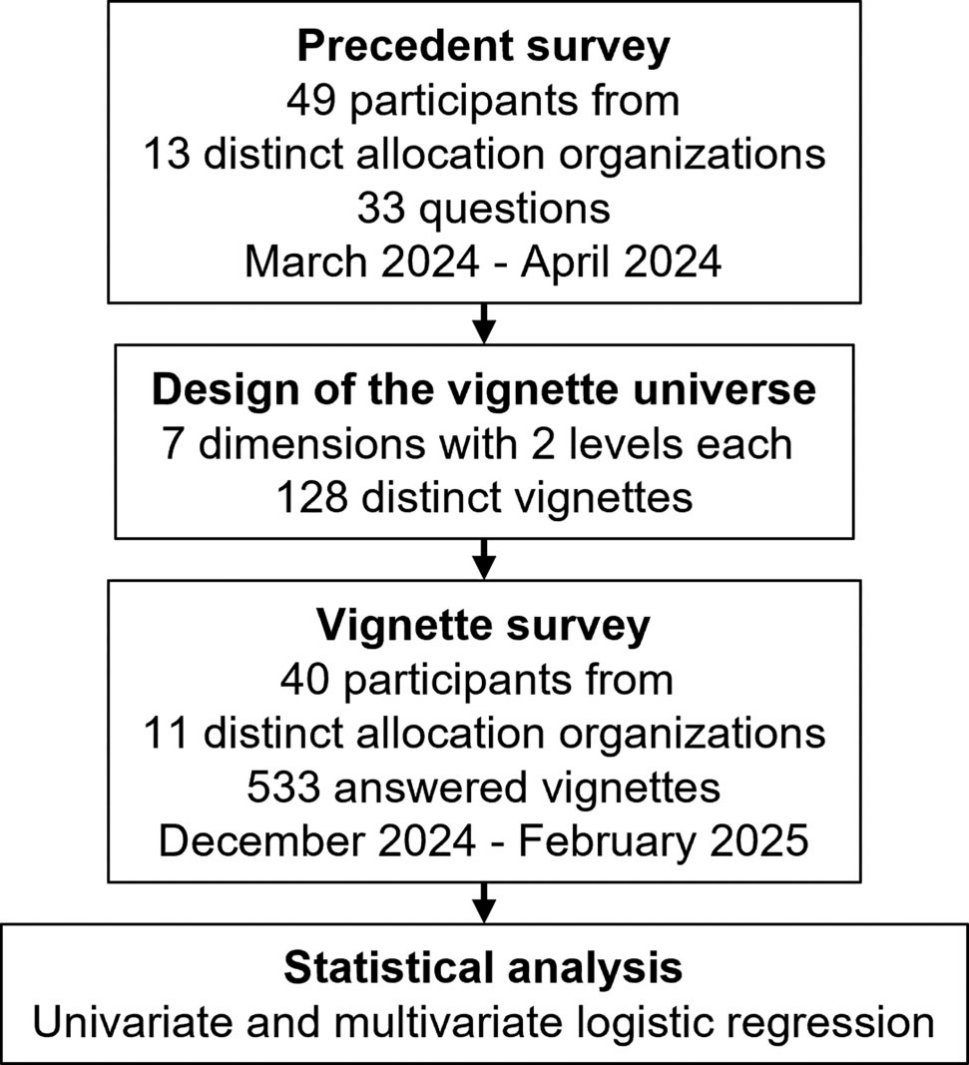
Process of survey conduction and vignette universe design. First, an initial precedent survey consisting of 33 questions about the decision-making process of a kidney offer to a pediatric kidney transplant recipient was conducted among 49 participants from 13 different distinct allocation organizations. Based on the results from the precedent survey, a vignette universe consisting of seven different dimensions with two different levels each resulting in 128 distinct vignette cases was designed in order to create a vignette survey. The vignette survey, which covered seven dimensions of a fictitious pediatric kidney offer [e.g. MMs on HLA loci, donor age, and donor kidney function (Table 1)], was conducted, resulting in 533 completed cases. These cases could then be statistically analyzed using uni- and multivariate mixed-effects logistic regression models.

We used a two-stage, web-based factorial vignette survey [16] (Fig. 1) to identify donor and recipient attributes that influence acceptance of deceased donor kidney offers for pediatric candidates. Vignette studies combine a general survey with short multidimensional situation descriptions to investigate people’s thinking and behavior patterns. These descriptions vary in predefined aspects (Table 1).

**Table 1:** Vignette universe.

Dimensions A–G		Levels a–g		Vignette text
A	HLA MMs on loci A, B, and C	a1 a2	≤3 ≥4	The number of MMs on HLA A, B, and C ___.
B	HLA MMs on loci DR, DQ	b1 b2	≤2 ≥3	On HLA DR, DQ there are __ MMs.
C	eGFR of an offered kidney (ml/min/1.73 m^2^)	c1 c2	≥50 ≤49	The eGFR of the offered kidney is __ ml/min/1.73 m^2^.
D	Donor age (years)	d1 d2	≤1 ≥2, ≤50	Donor age is __ years.
E	Recipient age (years)	e1 e2	≤1 ≥2	The recipient is __ years old.
F	Recipient: preemptive/dialysis	f1 f2	Preemptive dialysis	It is a preemptive transplantation./The recipient is a dialysis patient (hemodialysis or peritoneal dialysis).
G	Donor: sepsis/bacterial infection	g1 g2	Yes No	The donor has/does not have a history of sepsis or bacterial infection.

### Stage 1: precedent survey for elicitation of vignette attributes

In a first stage, we conducted an anonymous survey among pediatric nephrologists via the mailing server of the European Society for Paediatric Nephrology (ESPN). The survey asked about important factors influencing the decision to accept a kidney. This included 33 questions about topics such as cold ischemia time (CIT); mismatches (MMs) on HLA loci A, B, C, DR, and DQ; donor age; donor infection; and donor kidney function, i.e. estimated glomerular filtration rate (eGFR) (Supplementary Methods S1). The survey was conducted via SoSci Survey (Munich, Germany), which was hosted on MHH servers from March to April 2024.

### Stage 2: vignette survey design

Based on the results of the precedent survey, we identified the most relevant aspects for the vignette descriptions. From this multidimensional matrix, fictitious cases were compiled, with one parameter assigned to each dimension (Table 1). The number of vignette dimensions (Table 1, A–G) is seven, each with two levels (Table 1, a–g). This results in a vignette universe with 2 × 2 × 2 × 2 × 2 × 2 × 2 = 128 possible variations and therefore distinct vignettes. The following seven dimensions were included: (A) HLA MMs on loci A, B, C of ≤3 (a1)/≥4 (a2); (B) HLA MMs on loci DR, DQ of ≤2 (b1)/≥3 (b2); (C) eGFR of an offered kidney of ≥50 ml/min/1.73 m^2^ (c1)/≤49 ml/min/1.73 m^2^ (c2); (D) Donor age ≤1 year (d1)/2–50 years (d2); (E) Recipient age ≤1 year (e1)/≥2 years (e2); (F) Recipient status preemptive (f1)/on dialysis (f2); and (G) Donor with (g1)/without (g2) a history of sepsis or bacterial infection. The vignette text was used for each vignette case as described in Table 1 and Supplementary Methods S2.

We used an experimental factorial block design by assigning all possible vignettes to vignette sets. All participants answered one of eight sets, each consisting of 16 vignettes (8 × 16 = 128). This design ensured that the number of vignettes in each set was a multiple of the possible factor-level combinations, minimizing confounding effects by an even distribution of vignettes. The vignettes were distributed to the vignette sets using a matrix-based distribution strategy corresponding to a confounded factorial design (Supplementary Table S1). This allowed to minimize overlapping between factors at different levels, thereby reducing confounding effects. A set effect remained, but it only affected the higher levels of the vignette population. Due to the distribution of the vignettes, the two-way interactions were not confounded. The three- and four-way interactions were partially confounded (Supplementary Table S2). SoSci Survey (Munich, Germany), hosted on the MHH servers, was used for survey design and data collection. We report the web-survey components according to CHERRIES and designed the vignette experiment following best-practice guidance for experimental vignette studies [10, 12, 17].

### Stage 2: participants of the vignette survey

This study was approved by the Ethics Board of the Hannover Medical School, Hannover (11604_BO_K_2024). Each participant gave their informed consent to participate in this anonymous survey. The vignette sets were allocated to physicians from international centers for pediatric nephrology who are involved in accepting kidney offers for KTx in children. Vignette sets were randomly assigned to participants with equal distribution of the sampling of vignette sets to participants by SoSci Survey to almost equally assess every vignette (Supplementary Table S3). For each vignette of the assigned vignette set, every colleague decided whether to accept or decline the fictitious offer.

Data such as gender, center, allocation organization, and work experience were anonymously collected from these colleagues (Supplementary Methods S3). Recruitment took place between December 2024 and February 2025, via mailing lists of the following associations: European (ESPN, European Rare Kidney Disease Network), International (International Pediatric Transplantation Association), and national (Gesellschaft für Pädiatrische Nephrologie) societies/networks.

### Statistical analysis

For categorical variables, absolute and relative frequencies were presented. To evaluate the impact of vignette factors on the acceptance of a kidney offer, uni- and multivariate mixed-effects logistic regressions with a random intercept for respondent were conducted. Odds ratios (ORs), respective two-sided 95% confidence intervals, and two-sided *P*-values were provided. In the logistic regression, the probability of rejection of a kidney offer was modeled. An OR >1 means that this category of a vignette factor is associated with an increased odds of rejection of a kidney offer compared with the corresponding reference category of the same vignette factor. Statistical significance was considered achieved if a *P*-value was below .05. All analyses were performed using SAS Version 9.4 (SAS Institute Inc., Cary, NC, USA).

## RESULTS

### Stage 1: determination of vignette levels by a precedent survey

**Figure 2:**
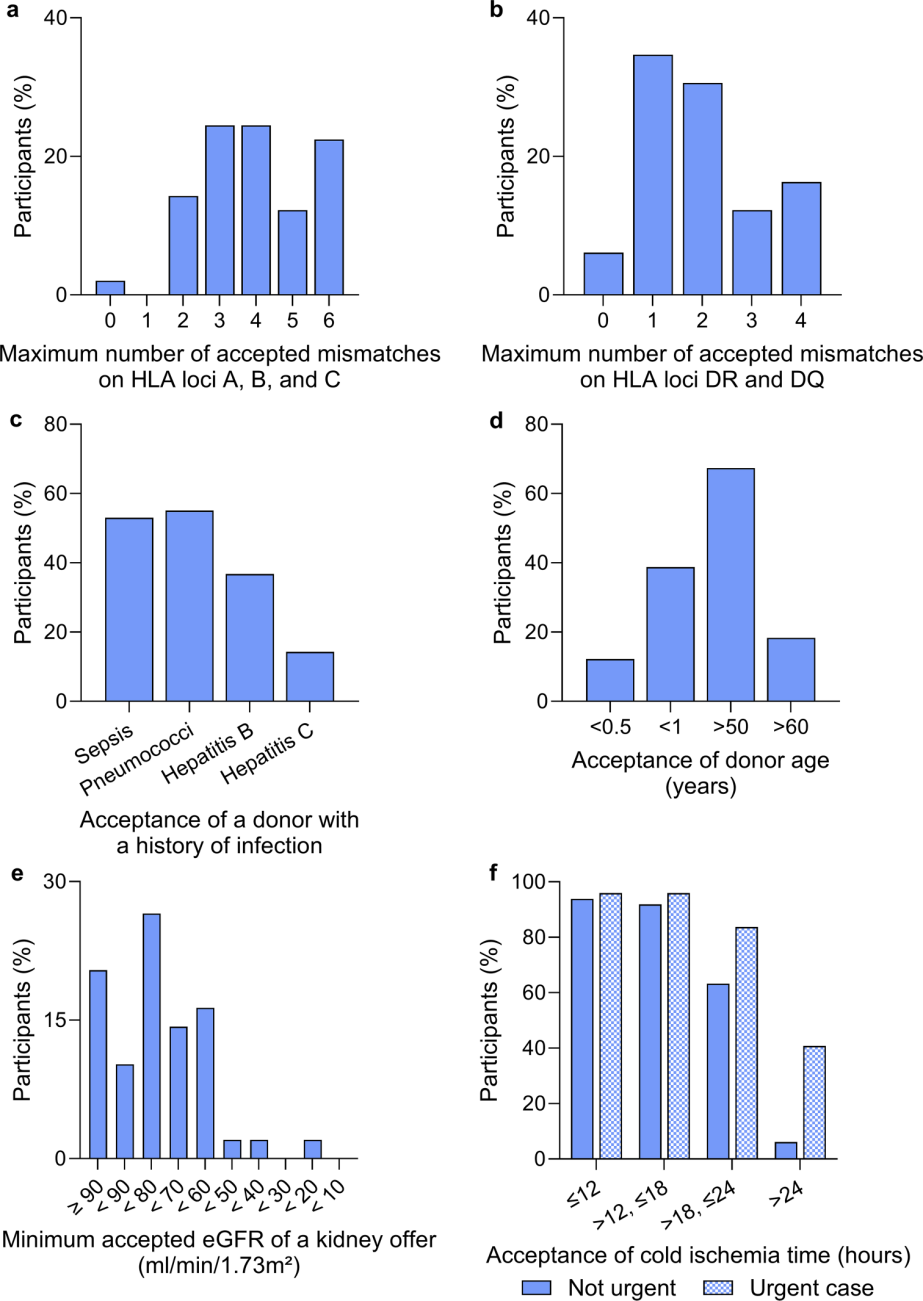
Precedent survey results. (a) Mostly a maximum of three (12/49, 25%) or four (12/49, 25%) MMs on HLA loci A, B, and C was accepted. Corresponding survey questions: ‘*Regarding ‘acceptable’ MM when a kidney is offered, what is the maximum total number of MM on HLA A, B, and C you would accept:*’. (b) The total number of acceptable MM on HLA DR and DQ was 1 (17/49, 35%) or 2 (15/49, 13%) at maximum in most cases. Corresponding survey questions: ‘*In our center, in order to accept a first kidney, transplant we allow the following maximum total number of MM on HLA DR and DQ:*’. (c) A history of sepsis or an infection with pneumococci in the donor was accepted in 55% (26/47) and 56% (27/48) of the cases, respectively, whereas a history of hepatitis C was mostly declined (42/49, 86%). Corresponding survey question: ‘*Would you accept a kidney from a donor with:*’. (d) Small donors aged <0.5 years were mostly declined (41/47, 87%). However, donors of >50 years were accepted in the majority of cases (33/49, 67%), whereas donors >60 years were mostly declined (40/49, 82%). Corresponding survey question: ‘*Regarding the age of the donor, you would accept a kidney from a donor who is aged:*’. (e) Most responses on the minimum accepted eGFR of an offered kidney ranged between ≥90 and <60 ml/min/1.73 m^2^ (43/49, 87.76%). Corresponding survey question: ‘*Regarding the donor’s kidney function, what is the minimal eGFR of an offered kidney that you would accept?*’. (f) CIT of >24 hours were declined in almost all cases (42/45, 93%); however, in urgent cases (marked in a hatched pattern) 43% (20/47) accepted a CIT of >24 hours. Corresponding survey questions: ‘*With regard to CIT, would you accept a deceased donor kidney with CIT:*’ and ‘*With regard to CIT, in clinically urgent cases would you accept a deceased donor kidney with CIT:*’.

A total of 49 participants from 13 allocation organizations completed the precedent survey (33 questions/items; Fig. 2 and Supplementary Methods S1). The presence of HLA A (46/47), HLA B (46/47), and HLA DR (48/48) loci was part of the decision-making process in at least 98% of cases, for HLA C and HLA DQ in 51% (22/43) and 69% (31/45) of cases, respectively. On HLA loci A, B, and C, the maximum total number of accepted MMs was in most cases 3 (25%, 12/49) or 4 (25%, 12/49) (Fig. 2a). The maximum total number of MMs accepted for DR and DQ was most commonly 1 (35%, 17/49) or 2 (31%, 15/49), four MMs were accepted in 16% (8/49) of cases (Fig. 2b). Kidneys from donors with sepsis or bacterial pneumococcal infections were accepted in 55% (26/47) and 56% (27/48) of cases, respectively (Fig. 2c). Small donors aged <0.5 years and <1 year were declined in 87% (41/47) and 60% (28/47) of cases, respectively. Donors aged >50 years were accepted in 67% (33/49) of cases, while those aged >60 years were mostly declined (40/49, 82%) (Fig. 2d). Regarding kidney function, a minimum eGFR >90 ml/min/1.73 m^2^ was required to accept a donor’s kidney in 22% (10/46) of cases. Notably, 17% (8/46) of the clinicians accepted a minimum eGFR of 50–60 ml/min/1.73 m^2^ (Fig. 2e). Regarding CIT, donor kidneys with CITs of <18 hours were accepted in >95% of cases. In urgent situations, CITs of 18–24 hours and >24 hours were accepted in 89% (41/46) and 43% (20/47) of cases, respectively (Fig. 2f). Many participants described the decision-making process as highly complex and emphasized the importance of recipient information. Based on the precedent survey the vignette universe shown in Table 1 was designed.

### Stage 2: participants of the vignette survey

In total, 31 participants responded completely and 9 partially to 1 of the 8 different vignette sets, each containing 16 vignettes. Thus, a total of 533 vignettes were answered, with an average of 3–6 responses for each of the 128 different vignette cases. Approximately 60% of the participants had >10 years of experience in pediatric KTx. More participants were female (24/40) than male (16/40). Participants came from ten European countries, Iran, and Canada and worked for eleven different allocation organizations, most commonly Eurotransplant, with 23 participants (23/40, 58%) (Table 2).

**Table 2:** Participant’s demographics.

**Background, *n* (%)**	
Resident/fellow in pediatric nephrology	6 (15%)
Consultant in pediatric nephrology (early career: 5–10 years in practice)	10 (25%)
Consultant in pediatric nephrology (> 10 years in practice)	24 (60%)
**Type of institution, *n* (%)**	
University hospital performing kidney transplants	37 (95%)
Nonuniversity hospital performing kidney transplants	1 (2.5%)
Private practice	1 (2.5%)
**Gender, *n* (%)**	
Female	24 (60%)
Male	16 (40%)
**Country of origin, *n* (%)**	
Belgium	1 (2.7%)
Canada	1 (2.7%)
Germany	19 (51%)
Iran	1 (2.7%)
Italy	4 (11%)
Netherlands	3 (8%)
Portugal	1 (2.7%)
Romania	1 (2.7%)
Spain	1 (2.7%)
UK	3 (8%)
France	1 (2.7%)
Switzerland	1 (2.7%)
**Allocation organization, *n* (%)**	
Eurotransplant—Austria, Belgium, Croatia, Germany, Hungary, Luxembourg, the Netherlands, and Slovenia	23 (58%)
France Transplant—France	2 (5%)
United Kingdom Transplant Support Services Authority—United Kingdom	4 (10%)
Swisstransplant—Switzerland	1 (2.5%)
Centro Nazionale Trapianti—Italy	4 (10%)
Organización Nacional de Trasplantes—Spain	1 (2.5%)
Romanian National Transplant Agency—Romania	1 (2.5%)
National Transplant Center—Israel	1 (2.5%)
Canada	1 (2.5%)
Health Ministry of Iran—Iran	1 (2.5%)
Portuguese Transplant Institute—Portugal	1 (2.5%)

*n*, number of participants

### Stage 2: external factors’ influence on the acceptance of a kidney offer

In univariate mixed-effects logistic regression, acceptance did not differ by experience [consultant in pediatric nephrology with >10 years of experience vs trainee/resident, OR 1.74 (0.82 to 3.70), *P* = .147] or gender [male vs female: OR 1.14 (0.62 to 2.10), *P* = .679] (Table 3). Decisions from centers within the Eurotransplant network were similar to those by centers in other allocation organizations (Not Eurotransplant vs Eurotransplant: OR 1.06 (0.57 to 1.96), *P* = .855) (Table 3).

**Table 3:** Impact of external factors with potential influence on the decision process.

	OR for rejection (95% confidence interval)^a^	*P*-value
**Allocation organization**		
Eurotransplant (reference)	–	–
Not Eurotransplant	1.06 (0.57 to 1.96)	.855
**Experience**		
Resident/fellow in pediatric nephrology (reference)	–	–
Consultant in pediatric nephrology (early career: 5–10 years in practice)	0.78 (0.34 to 1.83)	.574
Consultant in pediatric nephrology (>10 years in practice)	1.74 (0.82 to 3.70)	.147
**Gender**		
female (reference)	–	–
male	1.14 (0.62 to 2.10)	.679

^a^An OR >1 means that this category of a vignette factor is associated with an increased odds of rejection of a kidney offer compared with the corresponding reference category of the same vignette factor.

### Stage 2: factors impacting the acceptance of a kidney offer

Four of the seven vignette dimensions had a significant influence on the acceptance of a kidney offer in the multivariate mixed-effects logistic regression. This included vignette set and seven vignette dimensions as independent variables. These results were consistent with those of the univariate mixed-effects logistic regression (Table 4). Kidney offers with HLA MMs on HLA loci A, B, and C ≥4 [≥4 vs ≤3: OR 1.97 (1.17 to 3.32), *P* = .011] or on HLA loci DR and DQ ≥3 [≥3 vs ≤2: OR 14.24 (7.65 to 24.68), *P* < .001], donor eGFR ≤49 ml/min/1.73 m^2^ [≤49 vs ≥50: OR 9.92 (5.48 to 17.96), *P* < .001], or donor age ≤1 year [≥2, ≤50 vs ≤1: OR 0.45 (0.27 to 0.77), *P* = .003], were significantly more likely to be declined. In contrast, recipient age, whether the donor had sepsis or a bacterial infection, and whether the recipient was on dialysis or had undergone preemptive KTx, did not significantly influence the acceptance of a kidney offer. However, the results indicated a trend, albeit not significant, regarding offers from donors with a history of sepsis or a bacterial infection [without vs with history of sepsis/bacterial infection: OR 0.66 (0.39 to 1.10), *P* = 0.113] and recipients with preemptive KTx [on dialysis vs preemptive KTx: OR 0.60 (0.36 to 1.01), *P* = .055], suggesting that these were more likely to be declined. The data did not indicate a trend for recipient age (Table 4). Additionally, participants indicated for every answered vignette which of the seven dimensions was most influential from their point of view on their decision. Most commonly the number of MMs on HLA DR and DQ [Table 1, B; 32% (171/533)], and the eGFR of the offered kidney [Table 1, C; 28% (150/533)] were stated.

**Table 4:** Impact of vignette factors on the acceptance of a kidney offer.

	OR for rejection^a^ (95% confidence interval) *P*-value		
	All participants (univariate analysis)	All participants (multivariate analysis)	German participants (multivariate analysis)
**Vignette sets**			
Set 1 (reference)	–	–	–
Set 2	0.54 (0.17 to 1.75) .302	0.35 (0.06 to 2.15) .255	0.27 (0.03 to 2.98) .285
Set 3	0.52 (0.18 to 1.57) .247	0.37 (0.07 to 1.98) .245	0.89 (0.15 to 5.34) .899
Set 4	0.38 (0.12 to 1.21) .102	0.20 (0.03 to 1.17) .074	0.27 (0.04 to 1.79) .174
Set 5	1.03 (0.28 to 3.80) .965	0.99 (0.14 to 7.20) .995	2.37 (0.32 to 17.77) .399
Set 6	1.06 (0.31 to 3.69) .924	1.03 (0.16 to 6.61) .978	1.62 (0.19 to 13.79) .657
Set 7	1.15 (0.34 to 3.83) .824	1.13 (0.19 to 6.81) .892	4.53 (0.58 to 35.51) .149
Set 8	0.89 (0.28 to 2.77) .835	0.86 (0.15 to 4.79) .860	4.65 (0.35 to 62.26) .244
**HLA MMs on loci A, B, C**			
≤3 (a1, reference)	–	–	–
≥4 (a2)	1.57 (1.04 to 2.37) .033	1.97 (1.17 to 3.32) .011	2.05 (0.93 to 4.56) .076
**HLA MMs on loci DR, DQ**			
≤2 (b1, reference)	–	–	–
≥3 (b2)	7.55 (4.53 to 12.57) <.001	14.24 (7.65 to 24.68) <.001	45.25 (14.70 to 139.26) <.001
**eGFR of an offered kidney (ml/min/1.73 m^2^)**			
≥50 (c1, reference)	–	–	–
≤49 (c2)	5.16 (3.21 to 8.31) <.001	9.92 (5.48 to 17.96) <.001	6.20 (2.63 to 14.64) <.001
**Donor age (years)**			
≤1 (d1, reference)	–	–	–
≥2, ≤50 (d2)	0.59 (0.39 to 0.90) .014	0.45 (0.27 to 0.77) .003	0.52 (0.23 to 1.15) .105
**Recipient age (years)**			
≤1 (e1, reference)	–	–	–
≥2 (e2)	0.98 (0.65 to 1.47) .904	1.01 (0.60 to 1.70) .963	1.20 (0.55 to 2.65) .642
**Recipient: Preemptive/dialysis**			
Preemptive (f1, reference)	–	–	–
Dialysis (f2)	0.69 (0.46 to 1.05) .083	0.60 (0.36 to 1.01) .055	0.66 (0.30 to 1.46) .304
**Donor: Sepsis/bacterial infection**			
Yes (g1, reference)	–	–	–
No (g2)	0.75 (0.49 to 1.12) .160	0.66 (0.39 to 1.10) .113	0.69 (0.31 to 1.52) .354

^a^An OR >1 means that this category of a vignette factor is associated with an increased odds of rejection of a kidney offer compared with the corresponding reference category of the same vignette factor.

### Stage 2: subgroup analysis of German respondents

A multivariate analysis of the largest subpopulation (German respondents) was conducted. A total of 242 vignettes were answered by 19 German participants (Table 4). Of the seven factors, number of MMs on HLA DR and DQ ≥3 [≥3 vs ≤2: OR 45.25 (14.70 to 139.26), *P* < .001], and donor eGFR ≤49 ml/min/1.73 m^2^ [≤49 vs ≥50: OR 6.20 (2.63 to 14.64), *P* < .001], were significantly more likely to be declined. A trend toward declining a kidney offer was evident among patients with HLA MMs on HLA loci A, B, and C ≥4 [≥4 vs ≤3: OR 2.05 (0.93 to 4.56), *P* = .076], donors aged ≤1 year [≥2, ≤50 vs ≤1: OR 0.52 (0.23 to 1.15), *P* = .105], recipients with preemptive KTx [on dialysis vs preemptive KTx: OR 0.66 (0.30 to 1.46), *P* = .304], and donors with sepsis or bacterial infection [without vs with history of sepsis/bacterial infection: OR 0.69 (0.31 to 1.52), *P* = .354] (Table 4).

## DISCUSSION

In this study, we examined decision making for pediatric kidney offers using a fictitious factorial vignette survey to identify factors influencing acceptance. Donor and recipient characteristics were included based on a precedent survey (Table 1 and Fig. 1). Among clinicians from 11 allocation organizations, 4 of 7 factors significantly affected acceptance (higher HLA MMs on HLA loci A, B, and C or DR and DQ, donor eGFR, and donor age; Table 4). This experimental vignette design enabled realistic testing of multiple factors in a complex decision-making process. Recommendations on the design were taken into account [10, 18]. The strength of this methodology is that it can evaluate multiple influencing factors simultaneously [12]. Even when confounding higher-order interactions, the main effects are maintained [7, 14] (Supplementary Table S2).

A two-stage design with a precedent survey was used to determine the most relevant factors to include in the vignette universe, as well as potential cutoffs [16] (Fig. 1). There is no strict eGFR cutoff for accepting deceased donor kidneys, although a threshold of 60 ml/min/1.73 m^2^ has been recommended for living donors [19, 20]. In our precedent survey, most participants reported minimum acceptable donor eGFR values between ≥90 and <60 ml/min/1.73 m^2^ (Fig. 2), while eGFR <50 ml/min/1.73 m^2^ was accepted by only a minority, informing the design of Dimension C of the vignette universe (Table 1).

Eurotransplant’s primary allocation considers MMs at HLA loci A, B, and DR, with minimum typing at A, B, C, DR, and DQ [1]. In our precedent survey, most participants reported these loci as relevant for decision making (Fig. 2), supporting their inclusion in the vignette universe. As one to two MMs at DR and DQ were most commonly accepted, and DQ MMs have been linked to higher acute rejection rates [21, 22] despite not being part of primary allocation [1] , dimension B included ≤2 and ≥3 MMs at DR and DQ (Table 1).

As expected, kidney offers with ≥4 MMs on HLA loci A, B, and C as well as ≥3 MMs on HLA loci DR and DQ had a significantly higher chance of being declined in our vignette survey. An increased risk for acute rejections has been indicated for KTx with MMs on HLA DQ [21]. The risk of graft failure increases with the number of MMs on HLA A, B, and DR loci [23]. However, no strict cutoffs for acceptable MMs in the event of a kidney offer have been recommended thus far.

In the vignette survey, donors aged ≤1 year were significantly more likely to be declined. There are concerns about technical complexity, as specific microsurgical experience is required, and about complications such as thrombosis or vascular complications [24, 25]. However, KTx from small donors reportedly have good outcomes in experienced programs, reinforcing that surgical expertise and selection criteria should drive kidney offer acceptance. In previous reports, KTx from small donors had graft survival rates of 85% at 1- and 5-years post-transplantation [24], and 86.7%–93.3% in the first year for donors under 20 kg [25]. Previously, 3-year graft survival rate was 88.6% for kidneys from donors under 2 years, with no significant difference compared to donors aged 2–18 years [26].

Kidney offers from a donor with an eGFR below 50 ml/min/1.73 m^2^ were significantly more likely to be declined in our survey. Reportedly, for living donors under 50 years, an eGFR of ≥90 ml/min/1.73 m^2^ had a significantly better graft survival compared to those with a worse kidney function by eGFR [27]. A large registry study concluded that there was a higher risk of graft loss and mortality in KTx from deceased donors with acute kidney injury that did not recover, compared to those that partially or completely recovered [28]. However, graft survival rates of deceased donors with or without acute kidney injury were reported to be comparable in other studies [29, 30].

The vignette survey indicated a nonsignificant trend toward declining kidney offers for preemptive KTx recipients and donors with prior sepsis or bacterial infection. No trend for recipients aged ≤1 or ≥2 years was observed (Table 4). Previously, no significant difference in graft survival or complications was observed between children ≤/>15 kg [31]. The limited impact of donor infection history may reflect the low reported transmission risk of bloodstream infections [32–34], while the trend toward accepting offers for recipients on dialysis may relate to their higher cardiovascular risk compared with preemptive KTx recipients [35]. Evidence on graft survival remains conflicting, with single-center data showing no difference [6] and a 2022 meta-analysis favoring preemptive KTx [36]. Participant’s experience had no significant impact, possibly due to supervisory structures or clinic-specific guidance.

In conclusion, this international multicenter survey identified key factors influencing pediatric kidney offer acceptance, including HLA MMs, donor kidney function, and donor age. In this pilot study, a consistent pattern emerged, supporting further research toward explicit donor acceptance guidelines in children.

## LIMITATIONS

The statistical power of this study is limited by its small sample size. Further research on larger cohorts could increase statistical robustness. Geographical overrepresentation of German responses is a potential bias, as KTx rates in other European countries are higher [37]. To increase result representability, a replication survey could focus on a European representative response structure.

Although 533 vignettes were analyzed, the factorial design limited the number of dimensions. The impact of internationally differing factors [37, 38], such as probability of organ allocation, transplant access by differing waitlist inclusion criteria, rates of living transplantation, and national policies, along with morbidity of the recipient, including dialysis time, underlying disease, and complications, exceeded the scope of the vignette survey design.

Findings are based on fictious KTx offers. Future studies could assess real kidney offers to validate survey findings. Integrating significant factors into acceptance guidance may influence center-level decisions but could increase organ declines and waiting times; therefore, acceptance rates, waiting times, and outcomes should be closely monitored.

## Supplementary Material

sfag211_Supplemental_File

## Data Availability

Data not included in the manuscript or Supplementary material can be made available upon reasonable personal request to the corresponding author.
