# Supplementary material for: Factors influencing the acceptance of deceased donor kidney transplant offers in children—a vignette survey
Source: Clin Kidney J. 2026 Jun 22;19(8):sfag211. doi: 10.1093/ckj/sfag211 (PMC13437074; doi:10.1093/ckj/sfag211)
Supplement: Supplementary file 1 [file sfag211_supplemental_file.pdf]

Supplementary Material

Factors influencing the acceptance of deceased donor kidney transplant offers in children - a vignette survey

Leonie Greipel[1,2], Xiaofei Liu[3], Evgenia Preka[4,5], Lena Brunkhorst[1], Burkhard Tönshoff[6], Dieter Haffner[1], Nele Kanzelmeyer[1]

[1] Department of Pediatric Kidney, Liver, Metabolic and Neurological Diseases, Hannover Medical School, Hannover

[2] PRACTIS Clinician Scientist Program, Dean's Office for Academic Career Development, Hannover Medical School, Germany

[3] Institute for Biostatistics, Hannover Medical School, Hannover, Germany

[4] Université Paris Cité, INSERM U970 PARCC, Paris Institute for Transplantation and Organ Regeneration, Paris, France

[5] Pediatric Nephrology, Necker Enfants Malades University Hospital, Université Paris Cité, Paris, France

[6] Heidelberg University, Medical Faculty, Department of Pediatrics I, University Children's Hospital Heidelberg, Germany

Correspondence to: Leonie Greipel, Department of Pediatric Kidney, Liver and Metabolic Diseases OE 6720, Hannover Medical School, Carl-Neuberg-Str. 1, 30625 Hannover, Germany, Phone: +49 511 532 3601, Fax: +49 511 532 3911, E-mail: Greipel.leonie@mh-hannover.de

Supplementary Methods

Supplementary Methods S1. Questionaire of the precedent survey (stage 1).

1. What is your background?

- Resident/Fellow in paediatric nephrology
- Consultant in paediatric nephrology (early career: 5-10 years in practice)
- Consultant in paediatric nephrology (> 10 years in practice)

2. In what type of institution are you currently working?

- University hospital performing kidney transplants
- Non-university hospital performing kidney transplants
- Private hospital performing kidney transplants
- Other (please specify):

3. Considering the country in which you are employed, please indicate the organ allocation organisation to which you belong:

- Eurotransplant – Austria, Belgium, Croatia, Germany, Hungary, Luxembourg, the Netherlands and Slovenia
- Bulgarian Executive Agency for Transplantation – Bulgary
- Balttransplant – Estonia, Lithuania, Latvia
- Centro Nazionale Trapianti – Italy
- France Transplant – France
- Hellenic Transplant Organization – Greece
- Ministry of Health of the Republic of Montenegro – Montenegro
- National Transplant Center – Israel
- Nationalinis Transplantacijos Biuras – Lithuania
- Organ Donation Transplant Ireland – Ireland
- Organización Nacional de Trasplantes – Spain
- Poltransplant – Poland
- Romanian National Transplant Agency – Romania
- Scandiatransplant – Denmark, Finland, Iceland, Norway, Sweden and Estonia
- Slovak Transplant – Slovakia
- Stradiņa Klīniskā universitātes slimnīca – Latvia
- Swisstransplant – Switzerland
- Transplant Coordinating Center, Prague – Czech Republic
- Turkish Transplant Foundation – Turkey
- United Kingdom Transplant Support Services Authority – United Kingdom
- We have our own national kidney allocation organisation (not listed above).

4. In the context of receiving a kidney offer, this query aims to identify the institutional protocol governing the responsibility for accepting the kidney. The available options are:

Multiple answers are possible.

- Each fellow on call accepts or declines the kidney offer autonomously.
- A fellow's decision requires cross-validation with a consultant.
- Each consultant on call accepts or declines the kidney offer autonomously.
- A consultant's decision requires cross-validation with a second consultant.
- Our decision requires cross-validation with the on-call transplant surgeon.
- Other (please specify):

5. Does your paediatric kidney transplant centre follow a national uniform protocol regarding acceptance criteria for kidney offers intended for paediatric recipients?

- Yes
- No

6. Is it legal in your country to accept kidneys after brain death?

- Yes
- No

7. Is it legal in your country to accept kidneys after circulatory death?

- Yes
- No

8. Regarding HLA Loci, which of the following HLA Loci are considered in the process of accepting a kidney transplant offer in your center?

HLA A

- Yes
- No

HLA B

- Yes
- No

HLA C

- Yes
- No

HLA DR

- Yes
- No

HLA DQ

- Yes
- No

HLA DP

- Yes
- No

9. Are there other relevant loci which have not been included above?

10. Regarding “acceptable” mismatches (MM) when a kidney is offered on HLA A, B, C, what is the maxium total number of MM on HLA A, B, C you would accept:

- 0 MM
- 1 MM
- 2 MM
- 3 MM
- 4 MM
- 5 MM
- 6 MM

11. In our centre, in order to accept a first kidney transplant we allow the following maxium total number of mismatches (MM) on HLA-DR/-DQ:

- No MM
- 1 MM (either on DR and/or DQ)

- 2 MM (either on DR and/or DQ)
- 3 MM (either on DR and/or DQ)
- 4 MM

12. What is the cut-off value for a high virtual panel reactive antibodies (vPRA) in your centre? Above:

Please, indicate the cut-off value by moving the orange bar to the appropriate number of percentage.

13. In the case of having recipients with elevated virtual panel reacting antibodies (vPRA), you will accept donor kidneys of reduced quality e.g. reduced kidney function, very old donor, proteinuria, cardiovascular diseases, estimated prolonged cold ischaemia time:

- Yes
- No

14. With regard to cold ischemia time, would you accept a deceased donor kidney with:

Multiple answers are possible.

<= 12 hours

- Yes
- No

>12 and <= 18 hours

- Yes
- No

>18 and <= 24 hours

- Yes
- No

> 24 hours

- Yes
- No

15. With regard to cold ischemia time, in clinically urgent cases would you accept a deceased donor kidney with:

<= 12 hours

- Yes
- No

>12 and <= 18 hours

- Yes
- No

>18 and <= 24 hours

- Yes
- No

> 24 hours

- Yes
- No

16. Regarding the age of the donor, you would accept a kidney from a donor who is aged:

Multiple answers are possible.

< 0.5 years

- Yes
- No

< 1 year

- Yes
- No

> 50 years

- Yes
- No

> 60 years

- Yes
- No

17. Regarding the age difference between donor and recipient, would you accept a donor kidney if there was a significant age difference between them? More specifically,

Multiple answers are possible.

If the donor is > 40 years older than the recipient:

- Yes
- No

If the donor is > 50 years older than the recipient:

- Yes
- No

18. Regarding the donor's kidney function, what is the minimal eGFR of an offered kidney that you would accept?

- eGFR >= 90 mL/min/1.73 m^2
- eGFR < 90 mL/min/1.73 m^2
- eGFR < 80 mL/min/1.73 m^2
- eGFR < 70 mL/min/1.73 m^2
- eGFR < 60 mL/min/1.73 m^2
- eGFR < 50 mL/min/1.73 m^2
- eGFR < 40 mL/min/1.73 m^2
- eGFR < 30 mL/min/1.73 m^2
- eGFR < 20 mL/min/1.73 m^2
- eGFR < 10 mL/min/1.73 m^2

19. With regard to impaired diuresis, what is the minimum urine output in the last hour before transplantation for an offered kidney that you would accept?

- >= 0.5 mL/kg/h
- < 0.5 mL/kg/h
- < 0.3 mL/kg/h
- < 0.1 mL/kg/h
- Anuria

20. Would you accept a kidney from a donor with acute kidney injury (stage II-III)?

- Yes
- No

21. Would you accept a kidney from a deceased donor whose creatinine had been increased by more than 50% during the last admission?

- Yes
- No

22. Would you accept a kidney from a deceased donor whose creatinine had been increased by more than 100% during the last admission?

- Yes
- No

23. Would you accept a donor kidney from a donor who was on dialysis right before transplantation for:

Multiple answers are possible.

1 day

- Yes
- No

2 days

- Yes
- No

3 days

- Yes
- No

4 days

- Yes
- No

5 days

- Yes
- No

> 5 days

- Yes
- No

24. Would you accept a kidney from a donor who has had dialysis in the past?

- Yes
- No

25. Would you accept a kidney from a donor with sepsis?

- Yes
- No

26. Would you accept a kidney from a donor with bacterial infection with pneumococci (e.g. meningitis)?

- Yes
- No

27. Would you accept a kidney from a donor with a history of hepatitis B (HBs antigen negative, HBc antibody positive)?

- Yes
- No

28. Would you accept a kidney from a donor with a history of hepatitis C (HCV antibody positive)?

- Yes
- No

29. When the kidney transplant candidate is waiting for a second kidney transplant, HLA matching is very important:

- Yes
- No

30. When the kidney transplant candidate is waiting for a second kidney transplant, the age of the donor is very important:

- Yes
- No

31. When the kidney transplant candidate is waiting for a third kidney transplantat, HLA matching is very important:

- Yes
- No

32. When the kidney transplant candidate is waiting for a third kidney transplant, the age of the donor is very important:

- Yes
- No

33. Are there any factors in the kidney allocation process that we have not yet considered?

Supplementary Methods S2. The vignette text (stage2).

The following vignette text was used for each vignette case:

„During the shift, you will be offered a kidney for a pediatric patient from your transplant waiting list.

The number of mismatches on HLA A, B, and C ≤3 / ≥4.

On HLA DR, DQ, there are ≤2 / ≥3 mismatches.

The eGFR of the offered kidney is ≥50 / ≤49 ml/min/1.73m².

Donor age is ≤1 year / ≥2 and ≤50 years.

The recipient is ≤1 / ≥2 years old.

It is a preemptive transplantation. / The recipient is a dialysis patient (hemodialysis or peritoneal dialysis).

The donor has/does not have a history of sepsis or bacterial infection.

Would you accept the kidney offered for a kidney transplant in a pediatric kidney transplant recipient?”

Supplementary Methods S3. Survey questions on demographics answered by participants of the vignette survey (stage 2).

1. Are you or have you been involved in the decision process of accepting kidney offers for pediatric kidney transplant recipients?

- Yes
- No

2. Gender:

- female
- male
- non-binary
- prefer not to answer

3. In what type of institution are you currently working?

- University hospital performing kidney transplants
- Non-university hospital performing kidney transplants
- Private hospital performing kidney transplants
- Other (please specify):

4. Which country do you work in?

- Please, specify:

5. What is your background?

- Resident/Fellow in paediatric nephrology
- Consultant in paediatric nephrology (early career: 5-10 years in practice)
- Consultant in paediatric nephrology (> 10 years in practice)

6. Considering the country in which you are employed, please indicate the organ allocation organisation to which you belong:

- Eurotransplant – Austria, Belgium, Croatia, Germany, Hungary, Luxembourg, the Netherlands and Slovenia
- Bulgarian Executive Agency for Transplantation – Bulgary
- Balttransplant – Estonia, Lithuania, Latvia
- Centro Nazionale Trapianti – Italy
- France Transplant – France
- Hellenic Transplant Organization – Greece
- Ministry of Health of the Republic of Montenegro – Montenegro
- National Transplant Center – Israel
- Nationalinis Transplantacijos Biuras – Lithuania
- Organ Donation Transplant Ireland – Ireland
- Organización Nacional de Trasplantes – Spain
- Poltransplant – Poland
- Romanian National Transplant Agency – Romania
- Scandiatransplant – Denmark, Finland, Iceland, Norway, Sweden and Estonia
- Slovak Transplant – Slovakia
- Stradiņa Klīniskā universitātes slimnīca – Latvia
- Swisstransplant – Switzerland
- Transplant Coordinating Center, Prague – Czech Republic
- Turkish Transplant Foundation – Turkey

- United Kingdom Transplant Support Services Authority – United Kingdom
- We have our own national kidney allocation organisation (not listed above).
- Please, specify:

Supplementary Tables

Supplementary Table S1. Allocation of vignettes to vignette sets. Vignette dimensions (A-G) and levels (a-g) are defined by Table 1 as follows: (A) HLA mismatches on loci A, B, C ≤3 (a1) or ≥4 (a2); (B) HLA mismatches on loci DR, DQ ≤2 (b1) or ≥3 (b2); (C) estimated glomerular filtration rate (eGFR) of an offered kidney ≥50 mL/min/1.73 m² (c1) or ≤49 mL/min/1.73 m² (c2); (D) donor age ≤1 year (d1) or ≥2, ≤50 years (d2); (E) recipient age ≤1 year (e1) or ≥2 years (e2); (F) recipient status: preemptive (f1) or dialysis (f2); (G) donor history of sepsis or bacterial infection, yes (g1) or no (g2).

		**Vignette dimensions (A-G) with assigned vignette levels (a-g)**						
**Set Number**	**Vignette Number**	**A**	**B**	**C**	**D**	**E**	**F**	**G**
1	1	a1	b1	c1	d1	e1	f1	g1
	10	a1	b1	c2	d1	e1	f2	g1
	19	a1	b2	c1	d1	e2	f1	g1
	28	a1	b2	c2	d1	e2	f2	g1
	37	a2	b1	c1	d2	e1	f1	g1
	46	a2	b1	c2	d2	e1	f2	g1
	55	a2	b2	c1	d2	e2	f1	g1
	64	a2	b2	c2	d2	e2	f2	g1
	72	a1	b1	c1	d2	e2	f2	g2
	79	a1	b1	c2	d2	e2	f1	g2
	86	a1	b2	c1	d2	e1	f2	g2
	93	a1	b2	c2	d2	e1	f1	g2
	100	a2	b1	c1	d1	e2	f2	g2
	107	a2	b1	c2	d1	e2	f1	g2
	114	a2	b2	c1	d1	e1	f2	g2
	121	a2	b2	c2	d1	e1	f1	g2
2	2	a1	b1	c1	d1	e1	f2	g1
	11	a1	b1	c2	d1	e2	f1	g1
	20	a1	b2	c1	d1	e2	f2	g1
	29	a1	b2	c2	d2	e1	f1	g1
	38	a2	b1	c1	d2	e1	f2	g1
	47	a2	b1	c2	d2	e2	f1	g1
	56	a2	b2	c1	d2	e2	f2	g1
	57	a2	b2	c2	d1	e1	f1	g1
	71	a1	b1	c1	d2	e2	f1	g2
	78	a1	b1	c2	d2	e1	f2	g2
	85	a1	b2	c1	d2	e1	f1	g2
	92	a1	b2	c2	d1	e2	f2	g2
	99	a2	b1	c1	d1	e2	f1	g2
	106	a2	b1	c2	d1	e1	f2	g2
	113	a2	b2	c1	d1	e1	f1	g2
	128	a2	b2	c2	d2	e2	f2	g2
3	3	a1	b1	c1	d1	e2	f1	g1
	12	a1	b1	c2	d1	e2	f2	g1
	21	a1	b2	c1	d2	e1	f1	g1
	30	a1	b2	c2	d2	e1	f2	g1
	39	a2	b1	c1	d2	e2	f1	g1
	48	a2	b1	c2	d2	e2	f2	g1

	49	a2	b2	c1	d1	e1	f1	g1
	58	a2	b2	c2	d1	e1	f2	g1
	70	a1	b1	c1	d2	e1	f2	g2
	77	a1	b1	c2	d2	e1	f1	g2
	84	a1	b2	c1	d1	e2	f2	g2
	91	a1	b2	c2	d1	e2	f1	g2
	98	a2	b1	c1	d1	e1	f2	g2
	105	a2	b1	c2	d1	e1	f1	g2
	120	a2	b2	c1	d2	e2	f2	g2
	127	a2	b2	c2	d2	e2	f1	g2
4	4	a1	b1	c1	d1	e2	f2	g1
	13	a1	b1	c2	d2	e1	f1	g1
	22	a1	b2	c1	d2	e1	f2	g1
	31	a1	b2	c2	d2	e2	f1	g1
	40	a2	b1	c1	d2	e2	f2	g1
	41	a2	b1	c2	d1	e1	f1	g1
	50	a2	b2	c1	d1	e1	f2	g1
	59	a2	b2	c2	d1	e2	f1	g1
	69	a1	b1	c1	d2	e1	f1	g2
	76	a1	b1	c2	d1	e2	f2	g2
	83	a1	b2	c1	d1	e2	f1	g2
	90	a1	b2	c2	d1	e1	f2	g2
	97	a2	b1	c1	d1	e1	f1	g2
	112	a2	b1	c2	d2	e2	f2	g2
	119	a2	b2	c1	d2	e2	f1	g2
	126	a2	b2	c2	d2	e1	f2	g2
5	5	a1	b1	c1	d2	e1	f1	g1
	14	a1	b1	c2	d2	e1	f2	g1
	23	a1	b2	c1	d2	e2	f1	g1
	32	a1	b2	c2	d2	e2	f2	g1
	33	a2	b1	c1	d1	e1	f1	g1
	42	a2	b1	c2	d1	e1	f2	g1
	51	a2	b2	c1	d1	e2	f1	g1
	60	a2	b2	c2	d1	e2	f2	g1
	68	a1	b1	c1	d1	e2	f2	g2
	75	a1	b1	c2	d1	e2	f1	g2
	82	a1	b2	c1	d1	e1	f2	g2
	89	a1	b2	c2	d1	e1	f1	g2
	104	a2	b1	c1	d2	e2	f2	g2
	111	a2	b1	c2	d2	e2	f1	g2
	118	a2	b2	c1	d2	e1	f2	g2
	125	a2	b2	c2	d2	e1	f1	g2
6	6	a1	b1	c1	d2	e1	f2	g1
	15	a1	b1	c2	d2	e2	f1	g1
	24	a1	b2	c1	d2	e2	f2	g1
	25	a1	b2	c2	d1	e1	f1	g1
	34	a2	b1	c1	d1	e1	f2	g1
	43	a2	b1	c2	d1	e2	f1	g1
	52	a2	b2	c1	d1	e2	f2	g1
	61	a2	b2	c2	d2	e1	f1	g1
	67	a1	b1	c1	d1	e2	f1	g2
	74	a1	b1	c2	d1	e1	f2	g2
	81	a1	b2	c1	d1	e1	f1	g2
	96	a1	b2	c2	d2	e2	f2	g2

	103	a2	b1	c1	d2	e2	f1	g2
	110	a2	b1	c2	d2	e1	f2	g2
	117	a2	b2	c1	d2	e1	f1	g2
	124	a2	b2	c2	d1	e2	f2	g2
7	7	a1	b1	c1	d2	e2	f1	g1
	16	a1	b1	c2	d2	e2	f2	g1
	17	a1	b2	c1	d1	e1	f1	g1
	26	a1	b2	c2	d1	e1	f2	g1
	35	a2	b1	c1	d1	e2	f1	g1
	44	a2	b1	c2	d1	e2	f2	g1
	53	a2	b2	c1	d2	e1	f1	g1
	62	a2	b2	c2	d2	e1	f2	g1
	66	a1	b1	c1	d1	e1	f2	g2
	73	a1	b1	c2	d1	e1	f1	g2
	88	a1	b2	c1	d2	e2	f2	g2
	95	a1	b2	c2	d2	e2	f1	g2
	102	a2	b1	c1	d2	e1	f2	g2
	109	a2	b1	c2	d2	e1	f1	g2
	116	a2	b2	c1	d1	e2	f2	g2
	123	a2	b2	c2	d1	e2	f1	g2
8	8	a1	b1	c1	d2	e2	f2	g1
	9	a1	b1	c2	d1	e1	f1	g1
	18	a1	b2	c1	d1	e1	f2	g1
	27	a1	b2	c2	d1	e2	f1	g1
	36	a2	b1	c1	d1	e2	f2	g1
	45	a2	b1	c2	d2	e1	f1	g1
	54	a2	b2	c1	d2	e1	f2	g1
	63	a2	b2	c2	d2	e2	f1	g1
	65	a1	b1	c1	d1	e1	f1	g2
	80	a1	b1	c2	d2	e2	f2	g2
	87	a1	b2	c1	d2	e2	f1	g2
	94	a1	b2	c2	d2	e1	f2	g2
	101	a2	b1	c1	d2	e1	f1	g2
	108	a2	b1	c2	d1	e2	f2	g2
	115	a2	b2	c1	d1	e2	f1	g2
	122	a2	b2	c2	d1	e1	f2	g2

Supplementary Table S2. Confounding structure of the vignette sets for three- and four-way interactions. Letters refer to the 7 vignette dimensions.

Set	Three-way interactions: Confounded combinations	Four-way interactions: Confounded combinations
1	ADG, BEG, CFG	ABDE, ABCF, ACFG, ACDF, ACDG, ADFG, BCEF, BCFG, BCEG, BDEG, BEFG, CEFG, CDFG
2	ADE, ADF, ADG, BEF, CFG	ACDE, ADEF, ABDE, ABEF, ABCF, ACFG, ACDF, ACDG, ADFG, BCEF, BCFG, BCEG, BDEF, BEFG, CEFG, CDFG
3	ADE, BEG, CFG	ACDE, ADEF, ACFG, BCEF, BCFG, BCEG, BDEG, BEFG, CEFG, CDFG
4	ADE, ADF, ADG, BEF, CFG	ACDE, ADEF, ABDE, ABEF, ABCF, ACFG, ACDF, ACDG, ADFG, BCEF, BCFG, BCEG, BDEF, BEFG, CEFG, CDFG
5	ADG, BEG, CFG	ABDE, ABCF, ACFG, ACDF, ACDG, ADFG, BCEF, BCFG, BCEG, BDEG, BEFG, CEFG, CDFG
6	ADE, ADF, ADG, BEF, CFG	ACDE, ADEF, ABDE, ABEF, ABCF, ACFG, ACDF, ACDG, ADFG, BCEF, BCFG, BCEG, BDEF, BEFG, CEFG, CDFG
7	ADE, BEG, CFG	ACDE, ADEF, ACFG, BCEF, BCFG, BCEG, BDEG, BEFG, CEFG, CDFG
8	ADE, ADF, ADG, BEF, CFG	ACDE, ADEF, ABDE, ABEF, ABCF, ACFG, ACDF, ACDG, ADFG, BCEF, BCFG, BCEG, BDEF, BEFG, CEFG, CDFG

Supplementary Table S3. Frequency of responses per vignette.

Vignette Number	Frequency of responses	Percentage of total responses (%)
1	5	0.94
2	3	0.56
3	5	0.94
4	3	0.56
5	3	0.56
6	2	0.38
7	2	0.38
8	5	0.94
9	5	0.94
10	5	0.94
11	3	0.56
12	5	0.94
13	3	0.56
14	4	0.75
15	3	0.56
16	5	0.94
17	4	0.75
18	5	0.94
19	5	0.94
20	3	0.56
21	5	0.94
22	4	0.75
23	3	0.56
24	4	0.75
25	4	0.75
26	4	0.75
27	5	0.94
28	5	0.94
29	4	0.75
30	5	0.94
31	3	0.56
32	3	0.56
33	3	0.56
34	4	0.75
35	5	0.94
36	5	0.94
37	5	0.94
38	3	0.56
39	5	0.94
40	4	0.75
41	5	0.94
42	3	0.56
43	4	0.75
44	4	0.75
45	5	0.94
46	5	0.94
47	4	0.75
48	5	0.94
49	5	0.94
50	4	0.75

51	4	0.75
52	4	0.75
53	5	0.94
54	5	0.94
55	5	0.94
56	4	0.75
57	4	0.75
58	5	0.94
59	4	0.75
60	3	0.56
61	4	0.75
62	4	0.75
63	5	0.94
64	5	0.94
65	5	0.94
66	4	0.75
67	3	0.56
68	3	0.56
69	3	0.56
70	5	0.94
71	4	0.75
72	5	0.94
73	4	0.75
74	3	0.56
75	4	0.75
76	5	0.94
77	5	0.94
78	4	0.75
79	5	0.94
80	5	0.94
81	4	0.75
82	3	0.56
83	3	0.56
84	6	1.13
85	3	0.56
86	5	0.94
87	5	0.94
88	4	0.75
89	3	0.56
90	5	0.94
91	5	0.94
92	4	0.75
93	5	0.94
94	5	0.94
95	4	0.75
96	3	0.56
97	4	0.75
98	5	0.94
99	3	0.56
100	5	0.94
101	5	0.94
102	4	0.75
103	3	0.56
104	3	0.56

105	5	0.94
106	4	0.75
107	5	0.94
108	5	0.94
109	4	0.75
110	3	0.56
111	3	0.56
112	3	0.56
113	3	0.56
114	5	0.94
115	5	0.94
116	5	0.94
117	4	0.75
118	3	0.56
119	3	0.56
120	5	0.94
121	5	0.94
122	5	0.94
123	4	0.75
124	3	0.56
125	3	0.56
126	3	0.56
127	5	0.94
128	4	0.75
